# Canadian real-world evidence: observational 24-week outcomes for health care practitioner authorized cannabis

**DOI:** 10.1080/24740527.2025.2593253

**Published:** 2026-01-29

**Authors:** Brian Yang, Calvin Diep, Sonalben Thaker, Ted Jackson, Aly Lakhani, Carolina Landolt, Elena Pope, Irene Lara-Corrales, Henry Cortes Pradilla, Hai Chuan Yu, Gretchen Mae Gabriel, Rasheeda Darville, Ken Weisbrod, Keriann Tingling, Kiritpaul Nandra, Praveen Ganty, Joseph Fiorellino, Alexander Huang, Leeping Tao, Mandeep Singh, Peter Chung Tai, Peter Carlen, Karim Ladha, Hance Clarke

**Affiliations:** a Department of Anesthesia and Pain Management, Pain Research Unit, Toronto, Ontario, Canada; b Temerty Faculty of Medicine, University of Toronto, Toronto, Ontario, Canada; c Department of Anesthesiology and Pain Medicine, University of Toronto, Ontario, Canada; d Department of Emergency Medicine, Georgetown Hospital, Halton Healthcare, Georgetown, Ontario, Canada; e Department of Medicine, General Internal Medicine Division, Western Hospital, Toronto, Ontario, Canada; f Newmarket Pain Clinic, Newmarket, Ontario, Canada; g Independent Health Facility, Headwaters Health Care, Orangeville, Ontario, Canada; h Division of Dermatology, Department of Pediatrics, The Hospital for Sick Children, Toronto, Ontario, Canada; i Independent Health Facility, Interventional Pain Clinic Hospital Don Benito Villanueva de la Serena, Badajoz, Spain; j Department of Anesthesiology, Perioperative and Pain Medicine, University of Calgary, Calgary, Alberta, Canada; k Transitional Pain Service, Toronto General Hospital, University of Toronto, Toronto, Ontario, Canada; l Telus Health, Toronto, Ontario, Canada; m University Health Network, Toronto Western Hospital, Toronto, Ontario, Canada; n Division of Neurology, Toronto Western Hospital, Toronto, Ontario, Canada; o Division of Fundamental Neurobiology, Toronto Western Research Institute, Toronto, Ontario, Canada; p Department of Anesthesia, St. Michael’s Hospital, Toronto, Ontario, Canada

**Keywords:** Cannabis, cannabinoid medicine, real-world evidence, long term outcomes, chronic pain

## Abstract

**Background:**

With the increasing use of medical cannabis (MC), there is growing evidence suggesting that MC may be an effective therapeutic for chronic sleep, pain, and anxiety conditions. However, further evaluation is warranted to evaluate the heterogeneous patient outcomes of authorized cannabis treatment.

**Aims:**

To assess the effectiveness of authorized cannabis on pain, sleep duration, anxiety, and depression in patients presenting to clinics over a 6-month time period.

**Methods:**

This long-term prospective observational multicenter study utilized data from adult Canadian patients in the Medical Cannabis Real-World Evidence study. With physician guidance, patients were able to choose from Health Canada–verified MC products via a national pharmacy platform. Validated questionnaires were administered at Weeks 0, 6, 12, and 24 to assess pain interference (PROMIS, 6–30), pain score (NPRS, 0–10), sleep duration, anxiety (GAD-7, 0–21), depression (PHQ-9, 0–27), and quality of life (EQ-5D-3 L, 0–10). Outcomes were analyzed using descriptive statistics and generalized estimating equations models as both a per-protocol and intention-to-treat basis.

**Results:**

Improvements in pain, anxiety, depression, and QoL were observed from Baseline to Week 24. Decreases were observed in PROMIS Pain Interference – 4.6 (CI – 6.02 to – 3.17, *n* = 137), Numeric Pain Rating Scale – 1.19 (CI – 1.7 to −0.68, *n* = 137), General Anxiety Disorder-7 – 2.24 (CI – 3.5 to – 0.99, *n* = 139), Patient Health Questionnaire-9 depression −2.79 (CI – 4.29 to −1.3, *n* = 141), and EQ-5D-3 L – 0.56 (CI −0.96 to −0.16, *n* = 139).

**Conclusions:**

At Week 24, outcomes in chronic pain, anxiety, depression, and quality of life improved. Although 85% of patients in our study had an MC indication for pain, no outcomes met their minimal clinically significant thresholds at Week 24. These observations align with existing evidence, yet there remains some discrepancies in the current literature. Our findings highlight the need for future studies to characterize MC administration, dose, and specific product relationships.

## RÉSUMÉ

**Contexte :** Dans un contexte d’utilisation accrue du cannabis médical, des données probantes de plus en plus nombreuses indiquent qu’il pourrait constituer une option thérapeutique efficace pour les affections chroniques liées au sommeil, à la douleur et à l’anxiété. Toutefois, des évaluations supplémentaires sont nécessaires afin d’examiner l’hétérogénéité des résultats observés chez les patients recevant du cannabis médical autorisé.

**Objectifs :** Évaluer l’efficacité du cannabis autorisé sur la douleur, la durée du sommeil, l’anxiété et la dépression chez des patients se présentant en clinique sur une période de six mois.

**Méthodes :** Cette étude observationnelle prospective multicentrique de longue durée reposait sur des données provenant de patients adultes canadiens, recueillies dans le cadre de l’étude Medical Cannabis Real-World Evidence. Sous la supervision d’un médecin, les patients pouvaient choisir parmi les produits de cannabis médical autorisés par Santé Canada, accessibles au moyen d’une plateforme nationale de pharmacie. Des questionnaires validés ont été administrés aux semaines 0, 6, 12 et 24 afin d’évaluer l’interférence de la douleur (PROMIS, 6–30), le score de douleur (NPRS, 0–10), la durée du sommeil, l’anxiété (GAD-7, 0–21), la dépression (PHQ-9, 0–27) et la qualité de vie (EQ-5D-3L, 0–10). Les résultats ont été analysés à l’aide de statistiques descriptives et de modèles d’équations d’estimation généralisées, selon les analyses per protocole et en intention de traiter.

**Résultats :** Des améliorations de la douleur, de l’anxiété, de la dépression et de la qualité de vie ont été observées entre la visite initiale et la semaine 24. Des diminutions ont été observées pour l’interférence de la douleur mesurée par le PROMIS (−4,6; IC : −6,02 – −3,17; n = 137), le score de douleur mesuré par la Numeric Pain Rating Scale (−1,19; IC : −1,7 – −0,68; n = 137), l’anxiété mesurée par le GAD-7 (−2,24; IC : −3,5 – −0,99; n = 139), la dépression mesurée par le PHQ-9 (−2,79; IC : −4,29 – −1,3; n = 141), ainsi que le score de qualité de vie mesuré par l’EQ-5D-3L (−0,56; IC : −0,96 – −0,16; n = 139).

**Conclusions :** À la semaine 24, les résultats concernant la douleur chronique, l’anxiété, la dépression et la qualité de vie se sont améliorés. Bien que 85 % des patients de notre étude avaient une indication de cannabis médical pour le traitement de la douleur, aucun des résultats n’a atteint le seuil minimal de signification clinique à la semaine 24. Ces observations concordent avec les données probantes existantes, bien que certaines divergences subsistent dans la littérature actuelle. Nos résultats soulignent la nécessité de mener des études supplémentaires afin de mieux caractériser les modalités d’administration du cannabis médical, la posologie et les relations propres aux différents produits.

## Introduction

In 2024, over 180 000 Canadians were registered purchasers of medical cannabis (MC) from licensed MC vendors.^1^ Given its broadened legalization parameters, cannabis has been more frequently explored as an alternative medical therapeutic option by both patients and medical practitioners alike.^2–4^ Despite the known adverse effects that negatively impact cardiorespiratory and cognitive health with high doses of delta-9-tetrahydrocannabidol (THC), there remains promise in the therapeutic potential of MC that has yet to be fully explored.

Presently, the most commonly cited primary reasons for therapeutic cannabis use are chronic pain (28.4%), sleep (19%), stress (19%), anxiety (14.6%), and depression (9.6%).^5^ Cannabis use for chronic pain, the indication for 67% of MC prescriptions, has demonstrated analgesic properties and a potential for reducing opioid requirements.^5–7^ However, there remain variable and conflicting studies regarding the effectiveness of MC for sleep, stress, anxiety, and depression.^8–10^

Often, the variability of MC—the dosing, reliable cannabinoid proportions, and product—play a role in optimizing therapy. With over 160 characterized cannabinoids in the cannabis flower, active compounds such as terpenes and flavonoids, different growing and manufacturing components, and the alternative modalities of consumption, the effects and mechanisms of these factors remain poorly understood.^9^

Given the subjective experiences of individuals suffering from such chronic medical conditions, further attention should be directed to the potential long-term benefits of specific cultivars, and personalized dosing regimens and administration modalities.^11,12^ In this exploratory Medical Cannabis Real-World Evidence study (MCRWE), we sought to characterize 6-month patient outcomes related to chronic pain, sleep, stress, anxiety, depression, and quality of life when patients were given the opportunity to utilize a selection of Health Canada tested and verified Medical Cannabis health products.

## Methods

### Study population

Data from this study was obtained from the ongoing Medical Cannabis Real-World Evidence in Patient-Reported Outcomes Study, which began recruitment in 2020. This long-term prospective observational study was designed to assess the real-world practical application of MC in a cohort of adult Canadian patients authorized to use MC, using pre-defined, validated self-assessment scales. All adult patients with a prescription for MC and with a primary indication of pain (e.g., migraine, cancer, arthritis, multiple sclerosis), sleep disturbances (e.g., insomnia), epilepsy, and/or anxiety/depression were eligible for this study.

### Study recruitment and design

**Figure 1.**
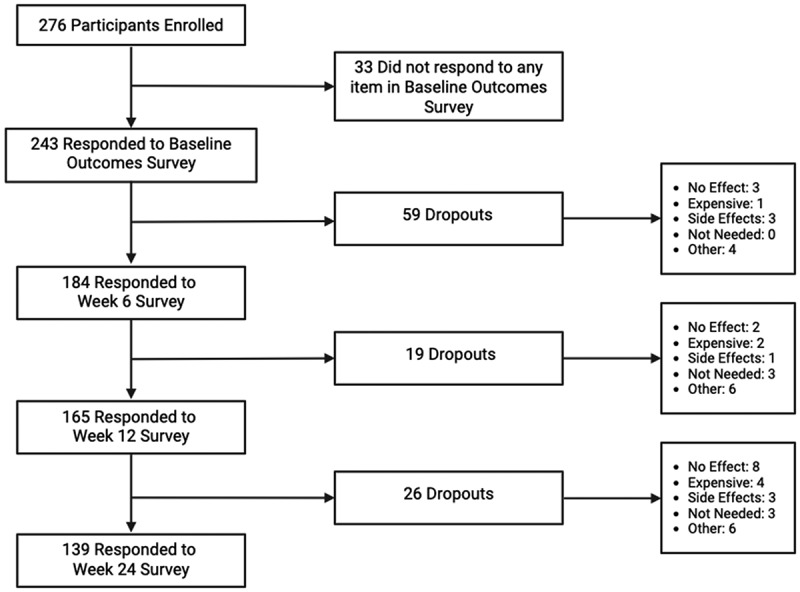
Patient response flow chart.

Patients and health care providers were informed of this study via posters and website advertisements. Patients could be recruited from any health care provider in Canada licensed to authorize MC once their patient consented to participate in Real World Evidence Medical Cannabis Study. Patients could also self-refer to the University Health Network Cannabinoid Therapeutics Center to register for the study. Once recruited, patients were administered several validated questionnaires, online via an e-mail line, at Baseline, Weeks 0, 6, 12 and 24. Reminders were conducted via telephone and patients were given 3 weeks to submit a response (Figure 1). Baseline questionnaires included demographic and medical history questions (e.g., previous/present diagnoses, medications); quality of life; and specific tools to assess pain, sleep, or anxiety/depression. Throughout the study and under physician guidance, participants were advised which cannabis products to use to treat their primary condition. The online ecosystem did enable the purchase of other MC products from the Cannabis by Shoppers Drug Mart offerings, and this was tracked. Under Shoppers Drug Mart, MC products also underwent an additional layer of testing by a third party to ensure that product components matched the label. This was to ensure that product labeling matched what was actually being consumed by patients.

This study was conducted in accordance with the protocol and all applicable laws and regulations including, but not limited to, good pharmacoepidemiology practices, and the ethical principles that have their origins in the Declaration of Helsinki and applicable privacy laws. Data protection and privacy regulations were strictly observed in capturing, forwarding, processing, and storing patient data. The study was reviewed and approved by Veritas (16560/ID-2485) and the University Health Network Research Ethics Board (20–5537). Informed consent was obtained online or written and faxed by each participant at the time of study registration.

### Primary outcomes

The primary outcomes for this observational exploratory study were patient pain interference, pain severity, anxiety, depression, sleep quality, and quality of life. These outcomes were collected at Baseline, Weeks 6, 12, and 24.

Patient pain interference outcomes were defined by the PROMIS Pain Interference—Short Form 6a scale. This scale consists of six questions that aimed to quantify the extent pain interfered with a patient’s engagement in social, cognitive, emotional, physical, and recreational activities.^13^ Each question was rated on a scale of 1 to 5. A score of 5 indicated pain interference was *very much*, 4 *quite a bit*, 3 *often*, 2 *a little bit*, and 1 *not at all*. The sum of scores from these six items was the total pain interference score, ranging from 6 to 30.

Pain severity experience was defined by the Numeric Pain Rating Scale (NPRS). Patients were asked to rate their subjective pain intensity now on a scale of 0 to 10.^14^ Here, 0 was defined as no pain and 10 was defined as pain as severe as the patient could imagine.

Sleep duration was self‑reported in pre‑specified hour bands. For analysis, we categorized participants into four groups: <5 h (very short sleep), 5–6 h (short sleep), 6–7 h (moderately short), and >7 h (reference sleep). These cut‑offs were selected based on prior epidemiologic evidence linking <6 h of sleep to adverse outcomes, and the established adult recommendation of 7–8 h (or 7–9 h) being optimal.^15–17^

Anxiety was measured using the General Anxiety Disorder 7-item (GAD-7). The GAD-7 consists of seven questions aimed to measure the severity of patient anxiety.^18^ Each question was an anxiety-related experience or problem rated on a scale of 0 to 3. A score of 3 indicated occurrence nearly every day, 2 indicated more than half of days, 1 indicated several days, and 0 being unsure. The sum of scores from these seven items was the total GAD-7 score, ranging from 0 to 21.

Depression was measured using the Patient Health Questionnaire (PHQ-9). The PHQ-9 is a validated screening instrument composed of nine items for identifying the presence and severity of symptoms of clinical depression in the past 2 weeks.^19^ Each item is scored from 0 to 3, with 0 indicating that the symptom was experienced on no days in the past 2 weeks, 1 if on several days, 2 if on more than half the days, and 3 on nearly every day. The sum of scores from these nine items was the total PHQ-9 score, ranging from 0 to 27.

Patient quality of life was measured using the EQ-5D-3 L. The EQ-5D-3 L is a 5-item questionnaire that is a generic measure of quality of life and self-reported health.^20^ Items include proficiency in mobility, self-care, engagement in usual activities, pain/discomfort, and anxiety/depression. Each item is scored from 0 to 2, with 0 indicating no decline, 1 if moderate, and 2 if severe or extreme. The sum of scores from these five items was the total EQ-5D-3 L score, ranging from 0 to 10.

### Baseline characteristics

Baseline characteristics such as age, gender, previous MC use, hypertension, diabetes, asthma, cancer, gastroesophageal reflux, arthritis, anxiety, depression, post-traumatic stress disorder (PTSD), tobacco cigarette smoking status, current use of opioids, NSAIDs, acetaminophen, and gabapentin/pregabalin, epilepsy/seizures, primary symptom improvement, the primary cannabinoid used CBD only, THC only and CBD/THC (%), and MC administration route were collected and described.

### Missing data and side effect symptoms

Patients could withdraw from the study at any time and had the opportunity to select the reasons for doing so (e.g., side effects, expensive, ineffective, etc.). Patients who withdrew from the study by not selecting a reason and were non-responsive to follow-ups were classified as Completely Lost to Follow-Up.

Patient data on side effects experienced, such as dizziness, nausea, headache, were also collected. Patients could select from a predetermined list of side effects common to cannabis consumption or freely input text if they had other unique symptoms.

### Data analysis

Baseline characteristics of the overall analytic cohort were summarized using descriptive statistics; mean ± standard deviation or counts with proportions were used, where appropriate. Each outcome variable of interest was also reported using mean ± standard deviation or counts with proportions, median, and interquartile range at each time point. Missingness in variables was reported as counts and proportions for all variables. To assess potential differences between patients who completed the 24-week follow-up and those who did not, we compared Baseline demographic, clinical, and cannabis-related variables between completers and non-completers. Patients were classified as dropouts if they had no outcome data available at Week 24. Continuous variables were summarized using means and standard deviations and compared using independent samples *t*-tests. Categorical variables were reported as counts and proportions and compared using chi-square tests. These analyses were conducted to identify whether any Baseline characteristics were associated with loss to follow-up, and results are reported in the Supplementary Materials.

For each outcome variable, a null model with time (Baseline, Week 6, 12, 24) as the explanatory variable was fitted to determine trends throughout the study period. All (excluding sleep) were fitted as both per protocol and intention-to-treat generalized estimating equations (GEE). Such GEEs are an extension of generalized linear models with several advantages when modeling data with repeated measures such as robustness to distributions of outcomes, robustness to mis-specified or unknown correlations between repeat measurements, and estimation of marginal (population-averaged) effects.^21^ Each outcome was modeled assuming a Gaussian distribution with an identity link function. To account for within-subject correlation over time, we specified an exchangeable working correlation structure. The GEE approach accommodates missing data under the assumption of missing completely at random. Because sleep duration was collected as four ordered categories and we aimed to test within-subject change between baseline and each follow-up, we applied the Stuart–Maxwell test on paired data (participants with non-missing sleep categories at both time points) to evaluate whether the distribution of sleep categories changes over time. Given missingness and potential sparsity in some cells, we report the paired sample size for each comparison.All statistical tests were two-sided with significance level defined at *p* < .05. All analyses were performed in RStudio Version 2024.04.2 + 764 (RStudio Team, Boston, MA), using *R* packages *tidyverse, readxl, gtsummary, lubridate, ggpubr, dplyr*, and *geepack* (fitting GEE models).^22–28^ Parameter estimates and 95% confidence intervals (CIs) were reported for all outcomes. Sample sizes were based on available data and no *a priori* power calculations were performed.

### Post hoc power analysis

Post hoc power calculations were conducted to assess the statistical power of the Week 24 sample compared to Baseline to detect clinically meaningful differences in key patient-reported outcomes over time. Assuming a two-sided alpha of 0.05, we used repeated measures ANOVA approximations to estimate the power to detect a medium effect size (Cohen’s *f* = 0.25) in primary outcomes (e.g., pain interference, sleep, anxiety, depression, and quality of life) across the four time points. Power calculations were conducted using the *pwr* package in *R*.

### Internal consistency (cronbach’s alpha)

Internal consistency of multi-item scales was assessed using Cronbach’s alpha at Baseline for PROMIS Pain Interference Short Form 6a (6 items), GAD-7 (7 items), and PHQ-9 (9 items). Analyses were conducted using the psych package in *R*.

## Results

A total of 276 individuals were enrolled in the MCRWE study at the completion of Shoppers Drug Mart participation in the industry. This cohort consisted of patients from seven different provinces (Ontario, Quebec, Newfoundland and Labrador, Manitoba, Saskatchewan, Alberta, and British Columbia), had a mean age of 48.4 ± 16.1 years, and 64.8% of the participants were female. Current indications for cannabis use included 85.2% for pain, 49.6% for sleep, and 36.7% reported anxiety and/or depression (Table 1). Full baseline characteristics are presented in Table 1. There were no significant differences in Baseline characteristics between dropout and non-dropout patients (Supplementary Table 16).

**Table 1.** Baseline characteristics of patients enrolled in the MCRWE study.

	Total study population (N = 276)	Missing (n)
Age, mean (SD), median, IQR	48.4 (16.1), 47, 36–60	9
Female Gender	173 (64.8%)	9
Previous MC Use	126 (46%)	19
Previous Recreational Cannabis Use	141 (53%)	10
Smoking Status (Tobacco):		14
Current	42 (16%)	
Previous	98 (37.4%)	
Never	122 (46.6%)	
^a^Concomitant Pain Medication Use		9
NSAID	81 (30.3%)	
Acetaminophen	104 (39%)	
Opioid	87 (32.6%)	
Gabapentinoid	71 (26.6%)	
^a^MC Indications:		
Pain	218 (85.2%)	20
Sleep	127 (49.6%)	
Anxiety/Depression	94 (36.7%)	
^b^Reported Symptom Improvement from Previous Cannabis Use:		10
Pain	97 (36.5%)	
Sleep	81 (30.5%)	
Anxiety	72 (27.1%)	
Depression	43 (16.2%)	

All values displayed are n (%) unless stated otherwise.

^a^Multiple selections allowed.

^b^Regardless of primary MC indication.

SD: standard deviation, GERD: gastroesophageal reflux, MC: medical cannabis, NSAID: non-steroidal anti-inflammatory, PTSD: post-traumatic stress disorder.

### Baseline characteristics

The majority of patients enrolled in the study had previously used MC, recreational cannabis, or both (Table 2). At Baseline (n = 276), indica (*n* = 41, 31.8%) and indica-sativa hybrids (*n* = 45, 34.9%) were the most previously used strains of MC by patients (Table 2). Generally, the MC THC and CBD concentrations (%) patients used prior to this study varied from 0% to over 25% (Table 2). For patients who have used MC prior to the study, oral capsules/oil were the most common route of administration (*n* = 70, 54.3%), followed by smoking/vaping (*n* = 57, 44.2%), and topical application (*n* = 2, 1.6%). Conversely, patients who used recreational cannabis prior to the study were smoking/vaping more frequently (*n* = 127, 89.4%) than consuming orally (*n* = 15, 10.6%). Full patient prior cannabis use characteristics are presented in Table 2.

**Table 2.** Characteristics of cannabis used by patients prior to enrollment.

	Total study population (N = 276)	Missing (n)
Any Cannabis Use	194	18
MC Only	52	
Recreational Only	67	
MC and Recreational	75	
No Previous Cannabis Use	64	
Type of MC Consumed		147
Indica	41 (31.8%)	
Sativa	10 (7.8%)	
Hybrid	45 (34.9%)	
CBD Only	19 (14.7%)	
Unsure	14 (10.9%)	
MC THC Concentration Used		147
0–2%	20 (15.5%)	
2–5%	6 (4.7%)	
5–10%	16 (12.4%)	
10–15%	12 (9.3%)	
15–20%	14 (10.9%)	
20–25%	24 (18.6%)	
>25%	18 (14%)	
Unsure	19 (14.7%)	
MC CBD Concentration Used		146
0–2%	14 (10.8%)	
2–5%	13 (10%)	
5–10%	14 (10.8%)	
10–15%	15 (11.5%)	
15–20%	10 (7.7%)	
20–25%	18 (13.8%)	
>25%	20 (15.4%)	
Unsure	26 (20%)	
MC Administration Route		147
Oral (Capsule/Oil)	70 (54.3%)	
Inhaled (Vape/Smoke)	57 (44.2%)	
Topical	2 (1.6%)	
Recreational Cannabis Administration Route		134
Oral (Capsule/Oil)	15 (10.6%)	
Inhaled (Vape/Smoke)	127 (89.4%)	

All values displayed are n (%) unless stated otherwise.

THC: tetrahydrocannabinol, CBD: cannabidiol.

Significant decreases in pain, sleep, anxiety, depression, and an increase in quality of life were observed from Baseline to Week 6 in all measured outcomes (Table 3). All differences were measured relative to Baseline.

**Table 3.** Mean patient study outcomes over time.

Total N = 276	Baseline	Week 6	Week 12	Week 24
Pain Interference (6–30) *Missing (n)* *Median, (IQR)*	21.95 (6.75) *33* *23, (18, 28)*	18.6a (6.81) *92* *18, (14, 24)*	18.7^a^ (7.06) *111* *19 (14, 24)*	17.54^a^ (7.22) *137* *18, (12, 24)*
Pain Severity (0–10) *Missing (n)* *Median, (IQR)*	5.19 (2.61) *33* *5, (3, 7)*	4.58^a^ (2.66) *92* *5, (2, 7)*	4.58^a^ (2.62) *111* *5, (3, 7)*	4.09^a^ (2.61) *137* *4, (2, 6)*
GAD-7 Total (0–21) *Missing (n)* *Median, (IQR)*	8.59 (6.26) *40* *8, (3, 13)*	6.42^a^ (5.81) *94* *5, (2, 10)*	6.68^a^ (5.85) *116* *5, (2, 10)*	6.74^a^ (6.03) *139* *5, (2, 10)*
PHQ-9 Total (0–27) *Missing (n)* *Median, (IQR)*	10.57 (6.96) *40* *9, (6, 16)*	7.76a (6.47) *94* *7, (2, 12)*	8.09^a^ (6.71) *116* *7, (3, 13)*	8.19^a^ (6.99) *141* *7, (2, 13)*
Quality of Life Index (0–10) *Missing (n)* *Median, (IQR)*	4.02 (1.88) *40* *4, (3, 5)*	3.46^a^ (1.9) *94* *3, (2, 5)*	3.46^a^ (1.93) *116* *3, (2, 5)*	3.5^a^ (1.91) *139* *4, (2, 5)*

Values displayed are mean (standard deviation).

^a^ p < 0.05 from Baseline

GAD-7: General Anxiety Disorder-7, PHQ-9: Patient Health Questionnaire-9.

### Pain outcomes

In per protocol analysis, at Baseline, patients had an average PROMIS Pain Interference score of 21.95 ± 6.75 (Table 3). This score decreased, on average, −3.26 at Week 6 (CI −4.08 to −2.44, *p* < .001), −2.98 at Week 12 (CI −3.95 to −2, *p* < .001), and −4.60 at Week 24 (CI −6.02 to −3.17, *p* < .001). Pain severity NPRS scores also saw a decrease from Baseline (5.19 ± 2.61), with the most dramatic changes occurring at Weeks 6 (−0.60, CI −0.93 to −0.27, *p* < .001), 12 (−0.56, CI −0.91 to −0.2, *p* < .005), and sustained into Week 24 (−1.19, CI −1.70 to −0.67, *p* < .001). In the intention-to-treat analysis model, all timepoints still had statistically significant, although attenuated, reductions (Supplementary Table 7, 8). In a subsequent analysis, we analyzed Pain Interference and NPRS differences across timepoints for patients who specifically had an MC indication for pain (*N* = 218) and removed the 15% who did not (*n* = 38). For this group, pain severity NPRS at Baseline was 5.57 ± 0.17 and at Week 24 it decreased −1.27 (CI −1.73 to −0.80, *p* < .001). Pain Interference at Baseline was 23.17 ± 0.42 and at Week 24 it decreased −4.89 (CI – 6.19 to −3.59, *p* < .001)

### Sleep outcomes

Among paired participants, the distribution of sleep categories shifted from Baseline to each follow-up. At Baseline, 28.4% (*n* = 67) of participants reported getting >7 hours of sleep per night, 36.9% (*n* = 87) reported 6–7 hours, 14% (*n* = 33) reported 5–6 hours, and 20.8% (*n* = 49) reported <5 hours. By Week 24, the distribution changed to 37.5% (*n* = 51) reporting >7 hours, 20.6% (*n* = 28) reporting 6–7 hours, 28.7% (*n* = 39) reporting 5–6 hours, and 13.2% (*n* = 18) reporting <5 hours (Table 4). These changes were statistically significant at *p* < .001 (X^2^=16.1) with an effect size of 0.21 (Supplementary Table 6).

**Table 4.** Patient sleep hours throughout the MCRWE study.

Total N = 276	Baseline	Week 6^a^	Week 12^a^	Week 24^a^
Sleep Hours				
Reference (>7) ΔBaseline	67 (28.4%)	93 (51.4%) +23%	61 (38.4%) +10%	51 (37.5%) +9.1%
Moderately Short (6–7) ΔBaseline	87 (36.9%)	43 (23.8%) -13.1%	30 (18.9%) -18%	28 (20.6%) -16.3%
Short (5–6) ΔBaseline	33 (14%)	19 (10.5%) -3.5%	48 (30.2%) +16.2%	39 (28.7%) +14.7%
Very Short (<5) ΔBaseline	49 (20.8%)	26 (14.4%) -6.4%	20 (12.6%)- 8.2%	18 (13.2%) -7.6%
Missing	40	95	117	140

All values displayed are n (%). P-values represent comparisons to Baseline.

^a^ p < 0.05 from Baseline

### Mental health outcomes

In addition, per protocol, patient GAD-7 anxiety scores improved from Baseline (8.59 ± 6.26). The largest absolute improvements occurred at Week 6 (−1.70, CI −2.37 to −1.03, *p* < .001) and Week 24 (−2.24, CI −3.50 to −0.99, *p* < .001). PHQ-9 depression scores also decreased relative to Baseline (10.57 ± 6.96) at all time points. At Week 6, patient PHQ-9 scores decreased on average −2.40 (CI −3.06 to −1.75, *p* < .001) and was maintained until Week 24 (−2.79, CI −4.29 to −1.30, *p* < .001). In the intention-to-treat analysis models, all timepoints still had statistically significant, although attenuated, reductions for both GAD-7 and PHQ-9 (Supplementary Table 9, 10)

### Quality of life outcomes

Finally, per protocol, patient EQ-5D-3 L quality of life scores also showed an overall improvement. At Baseline, the average EQ-5D-3 L was 4.02 ± 1.88. By Week 24, the average decreased −0.56 (CI −0.96 to −0.16, *p* < .001). In the intention-to-treat analysis model, all timepoints still had statistically significant, although attenuated, reductions (Supplementary Table 11)

### Adverse events and attrition

From Week 6 to 24, the number of patients reporting no side effects were 50 (27.2%) and 56 (40.3%), respectively. Of the total number of side effects reported at Week 6 and 24 (126, 106), the majority consisted of dry mouth (*n* = 24 at 19%, *n* = 26 at 24.5%), daytime sleepiness (*n* = 24 at 19%, *n* = 22 at 20.8%), and feeling high (*n* = 19 at 15.1%, *n* = 12 at 11.3%, Supplementary Table 14).

By Week 24 of this study, 139 (50.4%) provided a response and 137 (49.6%) patients dropped out completely (Figure 1). Of those who dropped out, only 13 cited no MC effect, seven cited financial burden, seven cited side effects, and six no longer needed MC as a reason for drop out. However, most dropouts did not cite a reason as they were unresponsive to follow-up.

### Post hoc power analysis

A post hoc power analysis was conducted using observed effect sizes and the final sample size at Week 24 to determine whether the study was sufficiently powered to detect changes in primary outcomes (Supplementary Table 12). The analysis assumed a two-tailed α level of 0.05. The study demonstrated excellent power (≥0.98) to detect mean changes in pain interference (effect size = −0.727, power = 1.000), numeric pain rating scale (effect size = −0.441, power = 0.999), PHQ-9 (effect size = −0.365, power = 0.981), and GAD-7 (effect size = −0.290, power = 0.898). In contrast, power was limited for EQ-5D-3 L (effect size = −0.198, power = 0.598) and sleep hours (effect size = −0.039, power = 0.072).

### Internal consistency

Internal consistency of the multi-item patient-reported outcome measures was assessed using Cronbach’s alpha in the Week 24 analytic sample (Supplementary Table 15). The PROMIS Pain Interference Short Form 6a demonstrated excellent reliability (α = 0.97). The GAD-7 and PHQ-9 scales also showed excellent internal consistency, with α values of 0.91 and 0.90, respectively.

## Discussion

In this report of the MCRWE study, we present outcomes for chronic pain, sleep, stress, anxiety, and depression from patients undergoing treatment with MC with physician guidance. Our findings suggest that MC may provide some benefits to patients using for pain, sleep, anxiety, and depression. These improvements appear to be most pronounced within the first 6 weeks.

Improvements in pain interference, as measured by the PROMIS Pain Interference scale, were observed as early as Week 6 and continued through Week 24. The average decrease of −4.60 points by Week 24 aligns with reductions reported in other studies of chronic pain management using cannabis-based therapies, but did not meet the minimal clinically important difference. For example, a systematic review by Mücke et al. (2018) demonstrated “much or very much improved” pain relief in up to 28.4% of patients with chronic non-cancer pain using cannabis-based interventions.^29^ However, they also found that 22.1% of individuals on a placebo control also reported the same quality of pain relief.^29^ Despite this, the review did find a clinically important decrease in pain intensity when using MC compared to placebo treatments. Another cross-sectional study by Bicket et al. (2023) also determined that over half of patients using MC for chronic non-cancer pain reduced their usage of opioids, non-opioids, and over-the-counter pain medications over a 12-month period, reinforcing the potential of MC as not only pain relief, but an effective alternative to opioids for some patient populations.^30^ The impressive NPRS pain score reductions in this study at 6 months, despite not meeting the minimal clinically important difference, also strongly suggest that further appropriately powered placebo controlled randomized trials are urgently needed.

Similarly, anxiety and depression, as measured by the GAD-7 and PHQ-9 scales, also improved over time, with the greatest reductions at Weeks 6 and 12. In total, there was an average decrease of −2.79 in PHQ-9 and –2.24 in GAD-7 scores by Week 24. However, neither meet their respective thresholds for minimal clinically important difference of 3.3 for PHQ-9 and 3.8 for GAD-7.^31,32^ Presently, there remains contradictory data on the directional and causal relationship between cannabis and anxiety.^33^ A review by Beletsky et al. (2024) did find some long-term studies that show lower levels of anxiety in cannabis users, however, it is also known that cannabis can increase anxiety in some patients. Therefore, the exact direction and strength of this relationship in a diverse patient group with diverse cannabis products is still mixed and unclear.^33^ Overall, the consistency between our findings and some existing literature warrants further examination of what MC regimens and doses may be beneficial to patients dealing with mental health conditions.

The changes in distribution of sleep hours, although significant at all Weeks 6, 12, and 24, still does not conclude directionality. However, we do see that a greater proportion of patients achieve > 7 hours of sleep by Week 24, and fewer report <5 hours of sleep. Directional analyses are an inherent limitation of Stuart-Maxwell tests.

However, this transient effect may reflect adaptation to MC or variability in patient-reported outcomes over time. Similar patterns were observed in a study by Tervo-Clemmens et al. (2023), where MC was found to initially improve sleep outcomes, but the effects could only be maintained at 12 weeks when participants increased their MC dosage.^34^ Such findings suggest that MC may be effective in producing short-term improvements in sleep quality.^34^ Quality of life, as measured by the EQ-5D-3 L, highlights the possible impact of MC on overall patient attitude, outlook, or tendency to their well-being. Reductions in EQ-5D-3 L scores at Week 6 and Week 24 highlight the potential of MC to sustain long-term quality-of-life improvements in patients. Studies by Aviram et al. (2021) and Haroutounian et al. (2016) also provide evidence for long-term symptom control using MC, with the ladder also reporting improved social and emotional disability scores.^35,36^ Another study by Arkell et al. (2023) also describes an association between MC use and quality of life improvements over 4 years for patients seeking treatment for non-cancer pain, cancer pain, insomnia, and anxiety.^37^

All analyses in this study were conducted on a per protocol basis, reflecting outcomes among patients who completed self-reported questionnaires at baseline and follow-up. This was deemed appropriate, given the real-world observational design and high attrition rate. As a sensitivity analysis, we also performed an intention-to-treat analysis using last observation carried forward and baseline observation carried forward methods, which also yielded significant differences in the same outcomes and timepoints as reflected in the per protocol analysis.

Although the observed improvements in pain, sleep, depression, and quality of life align with existing observational and clinical trial evidence, there remains some discrepancies in the literature.^33^ These may be due to differences in patient populations, MC formulations, or study designs. Furthermore, the wide variability in THC and CBD concentrations used among patients in our cohort highlights the need for future studies to better analyze the dose-response or strain-response relationships, if any. Future work should also explore whether primary medical cannabis indication or other patient-level variables moderate treatment response over time.

A key strength of the ongoing MCRWE study is the cost-effective use of validated self-assessment tools. This allows for the measurement of multiple patient-reported outcomes at different time points to provide insight into the temporal effects of MC use. Furthermore, this study is inclusive of a wide variety of demographic and medical subgroups that may benefit from MC, who may not otherwise meet the inclusion criteria for a clinical trial.^38,39^ Additionally, internal consistency for all multi-item scales used in the analysis was high, as assessed by Cronbach’s alpha: PROMIS Pain Interference (α = 0.97), GAD-7 (α = 0.91), and PHQ-9 (α = 0.90), supporting the reliability of these measures within this cohort. Furthermore, a post hoc power analysis based on observed effect sizes revealed that most primary outcomes—pain interference, pain intensity, GAD-7, and PHQ-9—were well powered (>0.89) to detect observed changes. In contrast, the sleep hours and EQ-5D-3 L quality-of-life outcomes were underpowered (power = 0.072 and 0.598, respectively), and thus any non-significant results for these variables should be interpreted cautiously. Another strength of this study is patients’ access to a variety of verified MC products of different strains, chemical composition, dosing, and regimens. As such, patients had the opportunity to engage in a higher level of personalized MC therapy under physician supervision, which had never been previously attempted in Canada. The diversity in study patients’ pre-existing medical histories, crossed with a variety of MC formulations, is useful for generating hypotheses about who is most likely to benefit from MC, under which conditions, for how long, and which MC products are most likely to do so.^40^

As all analyzed outcomes experienced improvement by at least Week 6, future MCRWE studies should identify prognostic factors of positive responses to medical cannabis, adverse event rates, and the types of cannabis products being purchased by Canadians. For example, exploring the differential effects of THC- and CBD-dominant products on specific outcomes may provide insight into tailoring MC therapies. Conversely, about 44% of participants used MC products with, or greater than, 15% THC, warranting further investigation on adverse side effects. Further investigation is needed to understand the long-term sustainability of benefits and/or adverse effects, particularly for sleep and quality-of-life outcomes.

There are several limitations in this study that must be acknowledged. First, the observational design precludes causal inferences about the effectiveness of MC. The relationships between MC use and outcomes such as pain or quality of life are complex and can be confounded by many demographic, social, medical, and behavioral factors that may not be included in the MCRWE data. For example, 46% and 51% of our cohort reported previous MC or recreational use, respectively, and we do not have data on last consumption prior to the study. Previous exposure to some form of cannabis may be a confounding variable that leads to different outcomes, compared to our 23% cannabis naive patients, and warrants further analysis. In addition, previous cannabis users may have already experienced some symptoms benefits, which potentially confounds or attenuates the benefits of the primary outcomes. Furthermore, data about simultaneous changes in other interventions for pain management such as health-related lifestyle changes and pain medications were not included. These factors would be important to document and understand to determine the true direct or indirect effect of MC on outcomes. The study also lacks a control group, making it difficult to disentangle the effects of MC from placebo effects, concurrent treatments, or potentially unauthorized use of recreational cannabis throughout the study. Last, approximately half (49%) of all patients dropped out or left incomplete self-reported data for outcomes in Week 24 and baseline cannabis use. This high rate of attrition is not unusual for MC studies. In other MC observational studies and retrospective analyses, attrition rates are up to 40–50% by weeks 12 and 24.^41–43^ To better understand the patterns of missing data at each follow-up timepoint, we categorized missingness into three groups: patients lost to follow-up (no data across all outcomes), patients who discontinued MC (self-reported reasons such as side effects or cost), and patients who selectively skipped one or more questionnaire items. These classifications provide a clearer picture of attrition and data completeness over time. These dropout rates could be due to lack of perceived efficacy, MC cost, and major side/adverse effects. These represent possible sources of bias if observed missingness is not random. Additionally, we examined whether Baseline demographic characteristics were associated with dropout status at Week 24. Compared to participants who completed follow-up, there were no significant differences between patients who completed Week 24 and those who dropped out prior. These results suggest that attrition may not be strongly related to demographic characteristics at Baseline, although unmeasured confounders may still play a role. For example, patients may be more likely to stay in the study if they have perceived cannabis benefit in the past. These warrant further investigation with larger patient samples. In future investigations, descriptive analyses based on baseline characteristics of dropouts can be compared to those who remained. Furthermore, statistical models such as mixed-effects models and multiple imputation could be employed to assess the influence of specific characteristics on dropout rates and estimate missing data. In addition, participants’ purchase records of MC products (provided by the vendor Shoppers Drug Mart Canada) will be used as an objective surrogate for usage throughout the study. However, this may introduce additional measurement bias as there would be no way for to track purchases of products from other authorized (or unauthorized) vendors.

## Conclusion

In conclusion, patients in the MCRWE study had improved scores with respect to a reduction in pain and pain-related disability, anxiety, depression, sleep, and overall quality of life. Often, the benefits of MC were maintained long-term into Week 24. Further data from the ongoing Medical Cannabis Real World Evidence study may offer additional insights into the usage of medical cannabis products and their potential benefits in the general population and inform dosing for future clinical trials focused on cohorts with specific medical conditions or indications.

## Supplementary Material

Supplemental Material
