# Supplementary material for: Canadian real-world evidence: observational 24-week outcomes for health care practitioner authorized cannabis
Source: Can J Pain. 2026 Jan 29;10(1):2593253. doi: 10.1080/24740527.2025.2593253 (PMC12867419; doi:10.1080/24740527.2025.2593253)
Supplement: Supplemental Material [file UCJP_A_2593253_SM4934.pdf]

Supplementary Table 1. Per protocol generalized estimating equation (GEE) models of pain interference measured using the total PROMIS Pain Interference score.

	β	95% Confidence Interval	p-value
**Time**			
Baseline	21.95	21.12, 22.79	
Week 6	**-3.26**	-4.08, -2.44	<0.001
Week 12	**-2.98**	-3.95, -2	<0.001
Week 24	**-4.6**	-6.02, -3.17	<0.001

Bolded values are statistically significant at $p<0.05$.

PROMIS-Pain Interference scale range: 6-30

Supplementary Table 2. Per protocol generalized estimating equation (GEE) models of pain severity measured using the Numeric Pain Rating Scale.

	β	95% Confidence Interval	p-value
**Time**			
Baseline	5.2	4.88, 5.53	
Week 6	**-0.6**	-0.94, -0.27	<0.001
Week 12	**-0.56**	-0.91, -0.2	<0.005
Week 24	**-1.19**	-1.7, -0.68	<0.001

Bolded values are statistically significant at $p<0.05$.

Numeric Pain Rating scale range: 0-10

Supplementary Table 3. Per protocol generalized estimating equation (GEE) models of anxiety severity measured using the total General Anxiety Disorder-7 score.

	β	95% Confidence Interval	p-value
**Time**			
Baseline	8.59	7.8, 9.37	
Week 6	**-1.7**	-2.37, -1.03	<0.001
Week 12	**-1.63**	-2.36, -0.9	<0.001
Week 24	**-2.24**	-3.5, -0.99	<0.001

Bolded values are statistically significant at $p<0.05$.

General Anxiety Disorder-7 scale range: 0-21

Supplementary Table 4. Per protocol generalized estimating equation (GEE) models of depression severity measured using the total Patient Health Questionnaire-9 score.

	β	95% Confidence Interval	p-value
**Time**			
Baseline	10.57	9.7, 11.43	
Week 6	**-2.4**	-3.06, -1.75	<0.001
Week 12	**-2.12**	-2.9, -1.34	<0.001
Week 24	**-2.79**	-4.29, -1.3	<0.001

Bolded values are statistically significant at $p<0.05$.

Patient Health Questionnaire-9 scale range: 0-27

Supplementary Table 5. Per protocol generalized estimating equation (GEE) models of quality of life measured using the total EQ-5D-3L score.

	**β**	**95% Confidence Interval**	**p-value**
**Time**			
Baseline	3.98	3.75, 4.21	
Week 6	**-0.46**	-0.66, -0.26	<0.001
Week 12	**-0.44**	-0.67, -0.22	<0.001
Week 24	**-0.56**	-0.96, -0.16	<0.001

Bolded values are statistically significant at $p<0.05$.

EQ-5D-3L scale range: 0-10

Supplementary Table 6. Stuart-Maxwell analysis of sleep hours categories.

Comparison	n-paired	X^2 (df=3)	p-value	Effect Size
Week 0 vs Week 6	163	27.2	**<0.001**	0.24
Week 0 vs Week 12	144	24.8	**<0.001**	0.24
Week 0 vs Week 24	125	16.1	**<0.005**	0.21

Sleep hours analysis was based on categories of <5 hours, 5-6 hours, 6-7 hours, >7 hours.

Supplementary Table 7. Last Observation Carried Forward generalized estimating equation (GEE) models of pain interference measured using the total PROMIS Pain Interference score.

	**β**	**95% Confidence Interval**	**p-value**
**Time**			
Baseline	21.60	20.80, 22.50	
Week 6	**-1.86**	-2.43, -1.30	<0.001
Week 12	**-1.88**	-2.54, -1.22	<0.001
Week 24	**-2.50**	-3.11, -1.89	<0.001

Bolded values are statistically significant at $p<0.05$.

Supplementary Table 8. Last Observation Carried Forward generalized estimating equation (GEE) models of pain severity measured using the Numeric Pain Rating Scale.

	**β**	**95% Confidence Interval**	**p-value**
**Time**			
Baseline	5.16	4.84, 5.47	
Week 6	**-0.34**	-0.57, -0.12	<0.001
Week 12	**-0.34**	-0.59, -0.10	<0.001
Week 24	**-0.55**	-0.78, -0.31	<0.001

Bolded values are statistically significant at $p<0.05$.

Supplementary Table 9. Last Observation Carried Forward generalized estimating equation (GEE) models of anxiety severity measured using the total General Anxiety Disorder-7 score.

	**β**	**95% Confidence Interval**	**p-value**
**Time**			
Baseline	8.27	7.52, 9.03	
Week 6	**-0.67**	-1.11, -0.23	<0.005
Week 12	**-0.76**	-1.24, -0.28	<0.005
Week 24	**-0.86**	-1.34, -0.38	<0.001

Bolded values are statistically significant at $p<0.05$.

Supplementary Table 10. Last Observation Carried Forward generalized estimating equation (GEE) models of depression severity measured using the total Patient Health Questionnaire-9 score.

	**β**	**95% Confidence Interval**	**p-value**
**Time**			
Baseline	10.20	9.36, 11.10	
Week 6	**-1.05**	-1.45, -0.65	<0.001
Week 12	**-1.09**	-1.57, -0.61	<0.001
Week 24	**-1.07**	-1.55, -0.59	<0.001

Bolded values are statistically significant at p<0.05.

Supplementary Table 11. Last Observation Carried Forward generalized estimating equation (GEE) models of quality of life measured using the total EQ-5D-3L score.

	**β**	**95% Confidence Interval**	**p-value**
**Time**			
Baseline	3.91	3.69, 4.14	
Week 6	**-2.1**	-0.33, -0.08	<0.005
Week 12	**-2.4**	-0.38, -0.10	<0.001
Week 24	**-2.3**	-0.37, -0.09	<0.005

Bolded values are statistically significant at p<0.05.

Supplementary Table 12~~3~~. Post-hoc power analysis between Baseline and Week 24.

Variable	Effect Size	Power
**Pain Interference**	-0.727	1.000
**Numeric Pain Rating Scale**	-0.441	0.999
**General Anxiety Disorder-7**	-0.290	0.898
**Patient Health Questionnaire-9**	-0.365	0.981
**EQ-5D-3L**	-0.198	0.598
**Sleep Hours**	-0.039	0.072

$\alpha = 0.05$

Supplementary Table 13. Number of reported symptom side effects per time point.

Symptom	Week 6	Week 12	Week 24
None	50	50	56
Heart Racing	8	8	4
Fainting	2	0	1
Feeling High	19	16	12
Dizziness	14	11	9
Daytime Sleepiness	24	21	22
Headache	13	17	8
Dry Mouth	24	29	26
Nausea	6	8	7
Paranoid	5	5	2
Other	11	14	15

Multiple survey selections allowed per patient.

Supplementary Table 14. Number of reported side effects per time point.

	Week 6 (N=184)	Week 12 (N=165)	Week 24 (N=139)
Total Side Effects Reported	126	129	106
No Side Effects	50	50	56
**Side Effect N (% of total reported)**			
Dry Mouth	24 (19%)	29 (22.5%)	26 (24.5%)
Daytime Sleepiness	24 (19%)	21 (16.3%)	22 (20.8%)
Feeling High	19 (15.1%)	16 (12.4%)	12 (11.3%)
Dizziness	14 (11.1%)	11 (8.5%)	9 (8.5%)
Headache	13 (10.3%)	17 (13.2%)	8 (7.5%)
Heart Racing	8 (6.3%)	8 (6.2%)	4 (3.8%)
Nausea	6 (4.8%)	8 (6.2%)	7 (6.6%)
Paranoid	5 (4%)	5 (3.9%)	2 (1.9%)
Fainting	2 (1.6%)	0 (0%)	1 (0.9%)
Other*	11 (8.7%)	14 (10.9%)	15 (14.2%)

Different side effect selections allowed per patient. One selection represents 1 or greater instance of the respective side effect.

*Indicates participants reporting any symptoms they have experienced not listed on questionnaires.

Supplementary Table 15. Internal consistency (Cronbach's Alpha) of multi-item validated questionnaires.

Scale	Cronbach's α
PROMIS Pain Interference	0.97
General Anxiety Disorder-7	0.91
Patient Health Questionnaire-9	0.90

Supplementary Table 16. Baseline characteristics of dropouts and non-dropouts in the MCRWE study.

	Dropouts (N=137)	Non-Dropouts (N=139)	p-value
**Age**, mean (SD)	47.2 (15.9)	49.6 (16.3)	>0.05
**Female Gender**	82 (63.1%)	91 (66.4%)	>0.05
**Previous MC Use**	60 (47.6%)	66 (50.4%)	>0.05
**Previous Recreational Cannabis Use**	77 (61.1%)	64 (49.2%)	>0.05
**Smoking Status (Tobacco):**			
Current	25 (19.4%)	17 (12.8%)	>0.05
Previous	52 (40.3%)	46 (34.6%)	>0.05
Never	52 (40.3%)	70 (52.6%)	>0.05
***Pain Medication Use**			
NSAID	41 (31.5%)	40 (29.2%)	>0.05
Acetaminophen	52 (40%)	52 (38%)	>0.05
Opioid	43 (33.1%)	44 (32.1%)	>0.05
Gabapentinoid	31 (23.8%)	40 (29.2%)	>0.05
***MC Indications:**			
Pain	105 (83.3%)	113 (86.9%)	
Sleep	56 (44.4%)	71 (54.6%)	>0.05
Anxiety/Depression	49 (38.9%)	45 (34.6%)	>0.05
****Reported Symptom Improvement from Previous Cannabis Use:**			

Pain	46 (35.4%)	51 (37.5%)	>0.05
Sleep	35 (26.9%)	46 (33.8%)	>0.05
Anxiety	36 (27.7%)	36 (26.5%)	>0.05
Depression	22 (16.9%)	21(15.4%)	>0.05

All values displayed are n (%) unless stated otherwise.

*Multiple selections allowed.

**Regardless of primary MC indication.

SD: standard deviation, GERD: gastroesophageal reflux, MC: medical cannabis, NSAID: non-steroidal anti-inflammatory, PTSD: post-traumatic stress disorder

No significant difference in any variable (p>0.05).
